# The Ancient Evolutionary History of Polyomaviruses

**DOI:** 10.1371/journal.ppat.1005574

**Published:** 2016-04-19

**Authors:** Christopher B. Buck, Koenraad Van Doorslaer, Alberto Peretti, Eileen M. Geoghegan, Michael J. Tisza, Ping An, Joshua P. Katz, James M. Pipas, Alison A. McBride, Alvin C. Camus, Alexa J. McDermott, Jennifer A. Dill, Eric Delwart, Terry F. F. Ng, Kata Farkas, Charlotte Austin, Simona Kraberger, William Davison, Diana V. Pastrana, Arvind Varsani

**Affiliations:** 1 Lab of Cellular Oncology, NCI, NIH, Bethesda, Maryland, United States of America; 2 Lab of Viral Diseases, NIAID, NIH, Bethesda, Maryland, United States of America; 3 Department of Biological Sciences, University of Pittsburgh, Pittsburgh, Pennsylvania, United States of America; 4 Department of Pathology, University of Georgia, Athens, Georgia, United States of America; 5 Animal Health Department, Georgia Aquarium, Inc., Atlanta, Georgia, United States of America; 6 Blood Systems Research Institute, San Francisco, California, United States of America; 7 Department of Laboratory Medicine, University of California, San Francisco, San Francisco, California, United States of America; 8 School of Biological Sciences, University of Canterbury, Christchurch, New Zealand; 9 Structural Biology Research Unit, Department of Clinical Laboratory Sciences, University of Cape Town, Cape Town, South Africa; 10 Department of Plant Pathology and Emerging Pathogens Institute, University of Florida, Gainesville, Florida, United States of America; Fred Hutchinson Cancer Research Center, UNITED STATES

## Abstract

Polyomaviruses are a family of DNA tumor viruses that are known to infect mammals and birds. To investigate the deeper evolutionary history of the family, we used a combination of viral metagenomics, bioinformatics, and structural modeling approaches to identify and characterize polyomavirus sequences associated with fish and arthropods. Analyses drawing upon the divergent new sequences indicate that polyomaviruses have been gradually co-evolving with their animal hosts for at least half a billion years. Phylogenetic analyses of individual polyomavirus genes suggest that some modern polyomavirus species arose after ancient recombination events involving distantly related polyomavirus lineages. The improved evolutionary model provides a useful platform for developing a more accurate taxonomic classification system for the viral family *Polyomaviridae*.

## Author Summary

Polyomaviruses are a family of DNA-based viruses that are known to infect various terrestrial vertebrates, including humans. In this report, we describe our discovery of highly divergent polyomaviruses associated with various marine fish. Searches of public deep sequencing databases unexpectedly revealed the existence of polyomavirus-like sequences in scorpion and spider datasets. Our analysis of these new sequences suggests that polyomaviruses have slowly co-evolved with individual host animal lineages through an established mechanism known as intrahost divergence. The proposed model is similar to the mechanisms through with other DNA viruses, such as papillomaviruses, are thought to have evolved. Our analysis also suggests that distantly related polyomaviruses sometimes recombine to produce new chimeric lineages. We propose a possible taxonomic scheme that can account for these inferred ancient recombination events.

## Introduction

Murine polyomavirus (MPyV) was discovered in the mid-1950s as a filterable infectious agent that could induce salivary tumors in experimentally exposed mice [2, 3]. It was quickly established that the virus is potently carcinogenic, causing many different types of tumors (Greek *poly* + *oma*) in various experimental systems. When the first primate polyomavirus, simian vacuolating virus 40 (SV40), was discovered as an abundant contaminant in early poliovirus vaccines that had already been administered to millions of individuals, it posed significant cause for alarm (reviewed in [4]). The ensuing rush to study the molecular biology of polyomaviruses provided a great wealth of insights into basic cell biology and the fundamental mechanisms of tumorigenesis (reviewed in [5]).

There is no conclusive evidence for productive transmission of SV40 among humans and it does not appear that the virus caused discernible disease in poliovirus vaccine recipients (reviewed in [6]). However, SV40 is closely related to human JC and BK polyomaviruses (JCV and BKV), both of which cause disease in immunosuppressed patients. JCV was discovered in a patient (initials JC) who was suffering from a lethal brain disease called progressive multifocal leukoencephalopathy (PML)[7]. BKV is rarely found in the brain, but causes serious kidney damage in up to 10% of kidney transplant recipients [8]. Conflicting reports suggest possible associations between JCV and BKV and additional human diseases, including prostate, colorectal, and kidney cancers [5, 9]. A more recently discovered human polyomavirus, Merkel cell polyomavirus (MCV), plays a key causal role in the development of a rare form of skin cancer, Merkel cell carcinoma [10]. Other recently discovered human polyomaviruses have been associated with a variety of disease states, ranging from thymic and lymphoid cancers to non-malignant skin dysplasias and vascular myopathy [11–14]. Efforts to discover additional human and animal polyomaviruses, and the conclusive establishment of further links to disease states, will undoubtedly remain highly active research areas for the foreseeable future.

It has been difficult to achieve consensus on the development of systems for taxonomic classification of polyomaviruses. This is regrettable, in the sense that the availability of a robust classification scheme could help guide researchers and clinicians toward an understanding of where to expect biological similarities and differences among established and newly discovered polyomavirus species. A key barrier to the development of a consensus taxonomic scheme has been the lack of a clear model for the evolutionary history of polyomaviruses. Approaches to this question have been limited by the fact that known polyomavirus species are derived from a restricted subset of terrestrial vertebrates. In this study, we report our discovery of polyomaviruses in several species of fish. Searches of shotgun genomics datasets also revealed previously unknown polyomavirus-like sequences in a surprisingly wide variety of additional animals, including insects and arachnids. We make use of these new, highly divergent polyomavirus sequences to develop an evolutionary model that might account for the interrelationships of extant polyomavirus species.

## Results

### Acquisition of divergent polyomavirus sequences

**Fig 1:**
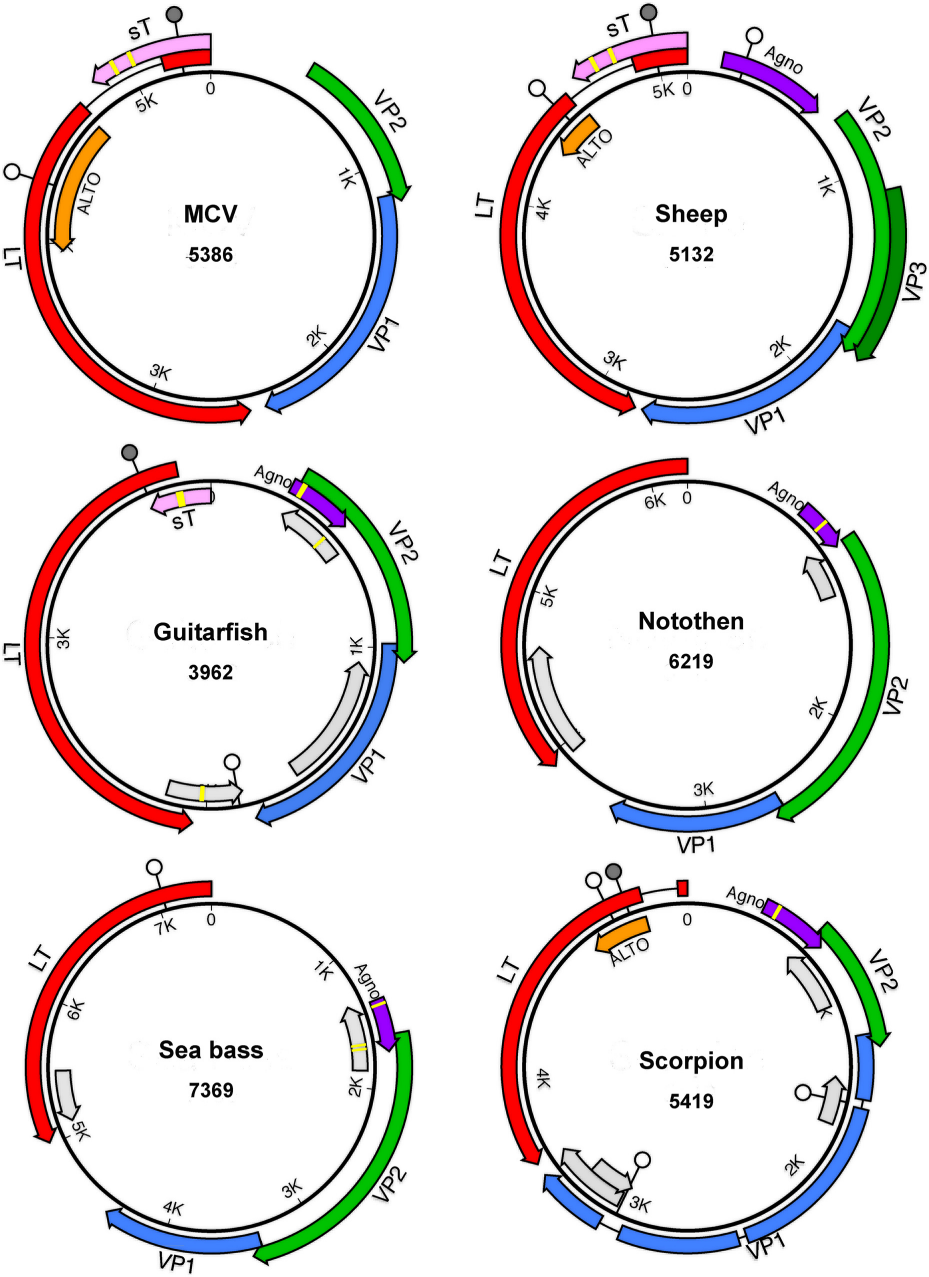
Predicted genetic organization of newly discovered polyomaviruses. Merkel cell polyomavirus (MCV) is shown as a well-studied reference species. The size of each genome (in basepairs) is listed below the species name. Large T antigen (LT) is indicated in red. Dark gray lollipops indicate the signature HPDKGG motif of the LT “DNAJ” domain (which appears to be missing from the sea bass and notothen polyomaviruses). White lollipops indicate LXCXE motifs, which are hypothetically involved in binding pRb and related tumor suppressor proteins. Each virus encodes a potential myristoylation signal that defines the N-terminus of the minor capsid protein VP2 (green). The VP2 of the supermarket sheep meat-associated virus encodes an internal MALXXΦ motif [1] that defines the N-terminus of a predicted VP3 minor capsid protein, while the other viruses do not. Predicted VP1 major capsid protein genes are shaded blue. ORFs found in the same general arrangement as previously described accessory proteins are also shown. These include small T antigen (sT, pink) Agnoprotein (purple), and the recently described ALTO (orange), which is overprinted in the LT +1 frame. Un-named ORFs of potential interest are shaded light gray. Yellow bars indicate hypothetical metal-binding motifs (CXCXXC or related sequences) observed in some of the predicted accessory proteins. Aside from MCV, for which expressed proteins have been experimentally confirmed, the predicted proteins are hypothetical and do not necessarily account for possible spliced transcripts.

In an effort to obtain more divergent polyomaviruses to use as reference points for understanding polyomavirus evolution, we sampled a variety of fish species. We have recently published a brief announcement describing the sequence of a polyomavirus found in samples of a perciform fish, black sea bass (*Centropristis striata*)[15]. In the current report, we present our discovery of another polyomavirus species found in a different perciform fish, the sharp-spined notothen (*Trematomus pennellii*) from McMurdo Sound (Ross Sea, Antarctica). The predicted genetic organization of these viruses is shown in Fig 1.

We also report a previously unknown polyomavirus species found in a giant guitarfish (*Rhynchobatus djiddensis*) suffering from papillomatous skin lesions. Guitarfish are members of the subclass Elasmobranchii, which includes sharks and rays. Elasmobranchs and bony vertebrates are thought to have diverged during the Cambrian period, about half a billion years ago [16]. Although the guitarfish polyomavirus encodes the characteristic polyomavirus arrangement of major open reading frames (Fig 1), its 3,962 bp genome is substantially smaller than the 4,697 bp genome of bovine polyomavirus 1, which had previously been the smallest known member of the family (see S1 File). To confirm that the virus directly infected the giant guitarfish (as opposed to an unknown environmental source), we performed in situ hybridization using a probe targeting the VP1 ORF. Hybridization signal was observed in small numbers of cells in resolving skin lesions (S1 Fig), confirming that the virus directly infects guitarfish.

We have recently reported the sequences of three polyomavirus species found in supermarket ground beef [17, 18]. In a follow-up effort using the same methods, we sampled supermarket ground turkey, American bison, and lamb. Although no polyomaviruses were found in the turkey or bison samples, a single previously unknown polyomavirus species was identified in the ground lamb (*Ovis aries*, sheep) meat sample. In light of recent scandals identifying traces of horse meat in supermarket ground beef products [19], the association of this virus with sheep should be considered tentative.

In GenBank keyword searches we noticed that a genomic DNA segment of a South African social spider (*Stegodyphus mimosarum*) had been annotated as having a patch of sequence similarity to polyomavirus LT (accession KK122585). The apparent endogenized “fossil” LT gene, which is integrated into a putative spider transcription elongation factor locus, was inferred to have one frameshift mutation and one nonsense mutation.

Polyomavirus protein sequences, including the novel fish polyomavirus LTs and a “resurrected” version of the social spider LT, were used to query translated nucleotide sequences in various NCBI databases. An additional fossil LT sequence was detected at a second locus in the social spider Whole Genome Shotgun (WGS) dataset. At least half a dozen fossil LT-like sequences could be detected in WGS entries for the common house spider (*Parasteatoda tepidariorum*). A short (170 bp) LT-like contig was identified in a third spider species, the Brazilian whiteknee tarantula (*Acanthoscurria geniculata*). Nearly a dozen transcripts with clear similarity to LT proteins were found in the Transcriptome Shotgun Assembly (TSA) datasets for two primitive insects, *Machillis hrabei* and *Meinertellus cundinamarcensis* (commonly called bristletails). More recently, some additional arthropod polyomavirus LT and VP1 transcripts have appeared in the TSA datasets for brown widow (*Latrodectus geometricus*) and cupboard spider (*Steatoda grossa*). Accession numbers for these newer sequences are listed in the “fragments” tab of S1 File.

Several polyomavirus-like sequences were also observed in TSA datasets for vertebrates, including a short VP1-like sequence in guineafowl (*Numida meleagris*), a short LT-like fragment in Carolina anole lizard (*Anolis carolinensis*), and an apparently complete set of spliced LT, VP1, and VP2 transcripts in the TSA dataset for dark-eyed junco (*Junco hyemalis*).

The most important discovery in the WGS database was a single contig (AXZI01204118) that appears to represent a nearly complete polyomavirus genome associated with Baja California bark scorpion (*Centruroides exilicauda*). Extension of the contig using individual reads from the parent Sequence Read Archive (SRA) datasets revealed two variants (~92% identity) of a circular non-integrated polyomavirus-like sequence. It thus appears that the individual animal used for the genome sequencing project happened to be productively infected with a polyomavirus. Although the complete, apparently episomal sequences show the usual organization of polyomavirus genomes, with highly divergent homologs of the standard LT and VP2 proteins (Fig 1), BLAST alignments using the inferred VP1 protein do not yield any convincing hits (E values >0.5).

### Structural modeling of divergent LT proteins

**Fig 2:**
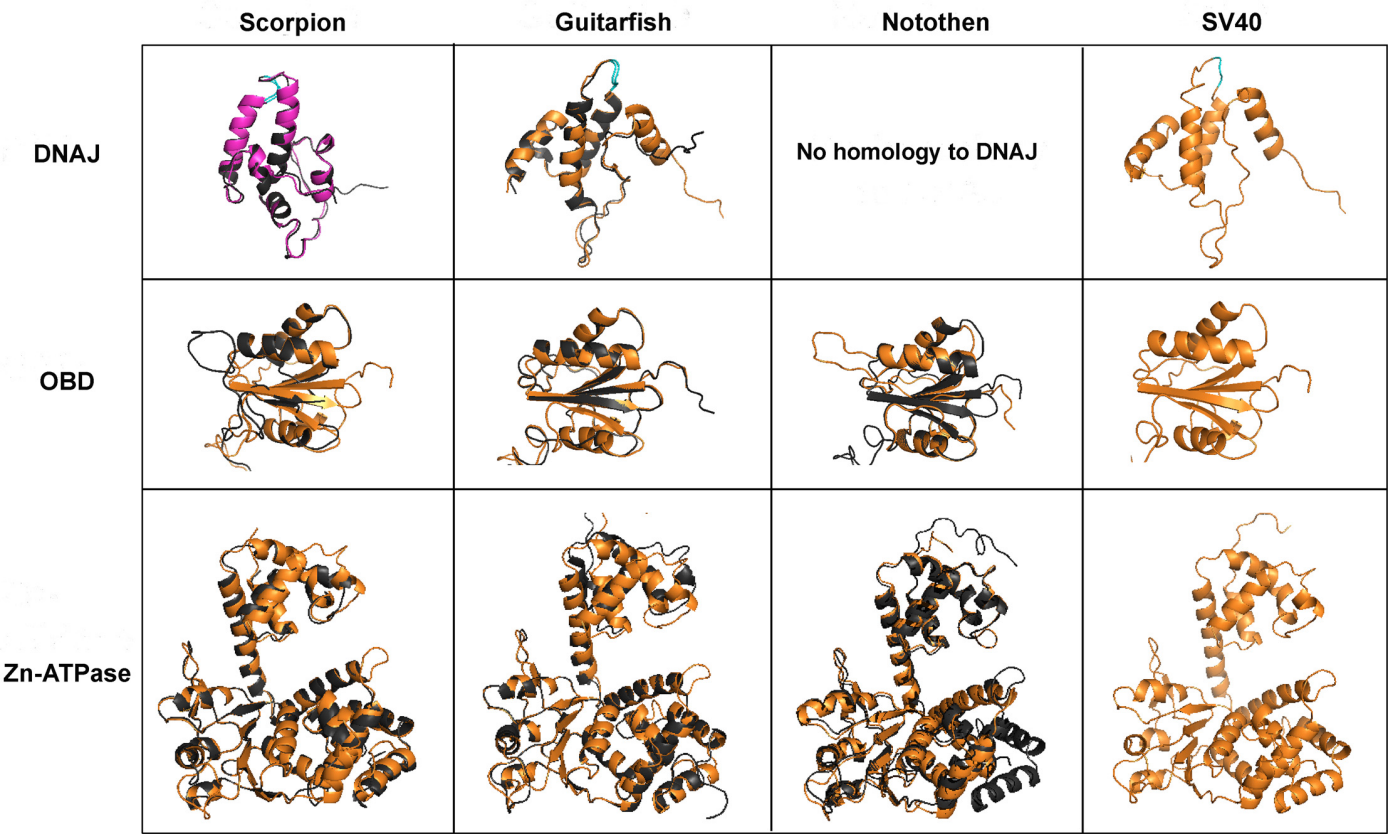
Structural modeling of LT proteins. The solved OBD-Zn-ATPase SV40 LT structure (PDB identifier 4GDF) was used as template for all OBD and Zn-ATPase domain models. The model of the guitarfish polyomavirus J domain was generated using the solved structure of the SV40 LT DNAJ domain (PDB identifier 1GH6) as template. For the DNAJ domain of scorpion polyomavirus LT, the best modeling template match is a *Thermus thermophilus* DNAJ protein (PDB identifier 4J7Z). The solved structure of the bacterial DNAJ is highlighted in magenta in the pairwise superimposition (top left). The LT proteins of the indicated polyomavirus species are shown in black. The known structures of SV40 LT domains are superimposed in gold. The conserved HPD motif of the DNAJ domain is positioned on the top and highlighted in cyan. The N-terminal domain of the notothen polyomavirus has no discernible structural similarity to known DNAJ structures.

Computer-based modeling was used to investigate the possible structural conservation of the apparent LTs of the new fish and arthropod polyomaviruses. SV40 LT is divided into discrete structural domains that are thought to exist in a “beads on a string” configuration (reviewed in [20]). The structures of individual LT domains have been solved [21, 22]. The modeled structures of the scorpion and fish LT origin binding domain (OBD), zinc finger domain, and ATPase domain each show a good fit with the known SV40 structures (Fig 2). A conservation map for the DNAJ and Zn-ATPase domains is shown in S2 Fig. These results confirm that the fish- and scorpion-derived sequences represent *bona fide* polyomavirus LT proteins.

LT proteins typically carry an N-terminal domain with sequence and structural similarity to cellular DNAJ chaperone proteins. The domain is defined by a hallmark linear motif, HPDKGG. The guitarfish and scorpion viruses share this motif, and the N-terminal domains of their LT proteins can readily be modeled onto known DNAJ structures (Fig 2). In contrast, the predicted sea bass and notothen polyomavirus LT proteins lack HPDKGG motifs. The two viruses are unique among known polyomaviruses in their apparent lack of any sequences that can be modeled onto known DNAJ structures. The novel N-terminal domains of the two perciform fish LT proteins share only about 25% similarity to one another, show no clear similarity to any other known proteins or protein structures, and are predicted to be unstructured. A possible explanation could be that the LT DNAJ domain is a common ancestral feature that was lost during development of the perciform fish polyomavirus lineage.

### Phylogenetic analysis of LT and VP1 proteins

A phylogenetic tree was constructed for the complete LT protein sequences of examples of all currently known polyomavirus species and sub-genomic fragmentary sequences available prior to November, 2015. The phylogenetic analyses also included putative LT protein sequences found in a pair of viral species that cause carcinomatosis in an Australian marsupial, the western barred bandicoot (*Perameles bougainville*). The bandicoot viruses appear to have arisen after recombinant chimerization involving an unidentified polyomavirus and a member of a known group of marsupial-tropic papillomaviruses [23, 24]. The apparently chimeric viruses encode a polyomavirus LT-like gene on one strand and genes for papillomavirus-like L1 and L2 capsid proteins on the other strand.

Like the bandicoot viruses, a different apparently chimeric virus called Japanese eel endothelial cells-infecting virus (JEECV) encodes a protein with typical LT features, including an N-terminal DNAJ-like sequence domain [25]. A similar virus has recently been discovered in Taiwanese marbled eels [26]. Aside from the clear 2.1 kb LT gene, the remaining ~13 kb of the JEECV genome bears little similarity to sequences in GenBank. It thus appears that JEECV and the marbled eel virus arose through recombination between a bony fish-associated polyomavirus and a member of another DNA virus family that remains unidentified.

**Fig 3:**
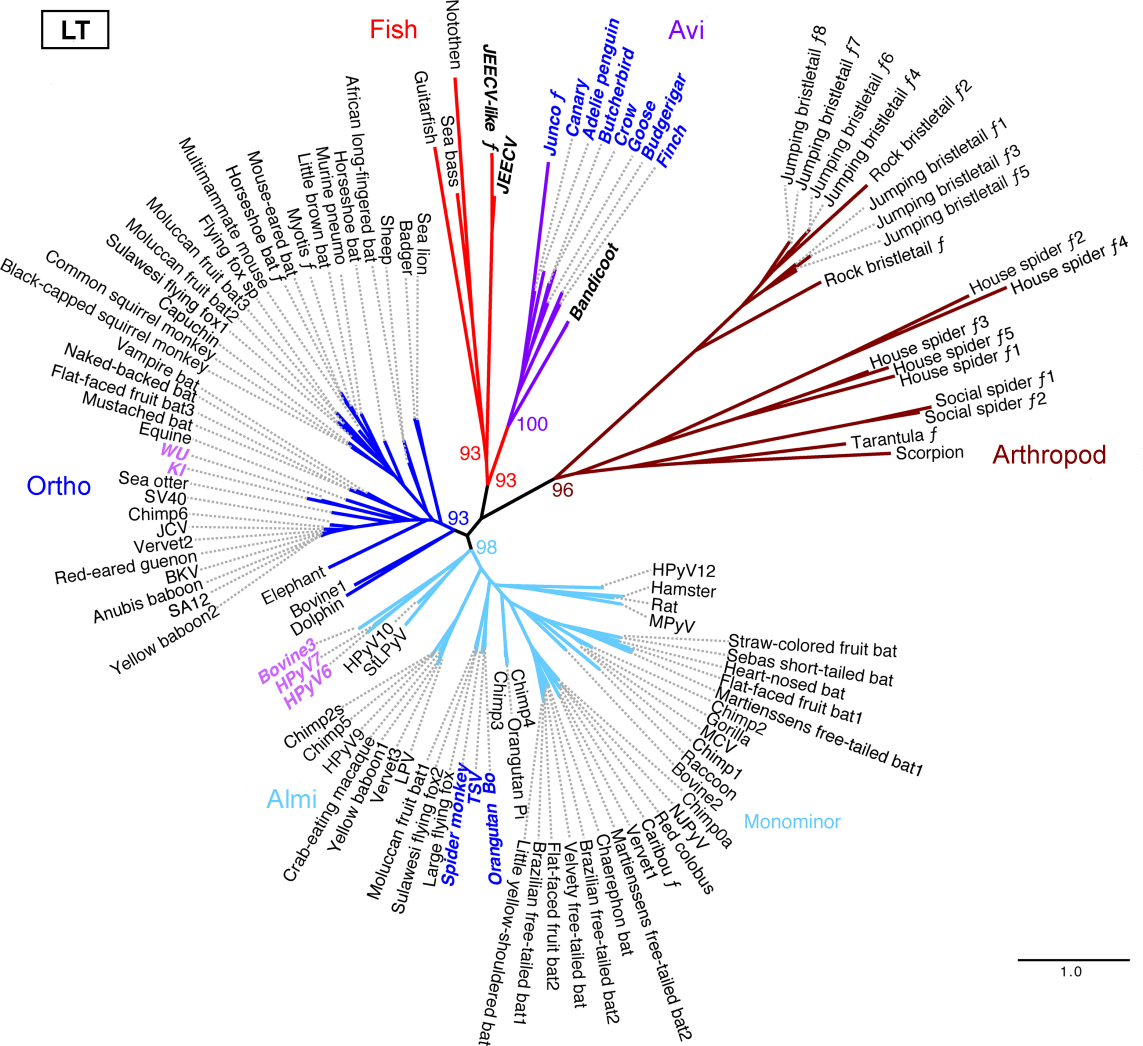
Midpoint-rooted phylogenetic tree for polyomavirus Large T antigen (LT) protein sequences. Species with different clade affiliations in VP1 analyses (Fig 4) are indicated in colored bold oblique text. The script ƒ character indicates fragmentary (sub-genomic) sequences. A key to species nicknames, genetic characteristics, and accession numbers is provided in S1 File. Percent bootstrap values are indicated for selected nodes. A FigTree file containing detailed bootstrap values is provided as S2 File. Scale bar shows one substitution per site.

Phylogenetic analysis of LT proteins (Fig 3) shows distinct clades corresponding to fish- and arthropod-associated sequences, as well as the previously recognized mammalian Ortho and Almi clades [27, 28]. Avian and bandicoot LT sequences together occupy a distinct clade. The appearance of the bandicoot virus LT protein sequences within this clade suggests that polyomaviruses with Avi-like early regions may infect modern marsupials. The avian and bandicoot LT proteins occupy a larger super-clade that loosely includes the newly identified fish-associated LT sequences. The LT protein sequences of the fish-associated polyomaviruses form a distinct clade that includes JEECV LT.

**Fig 4:**
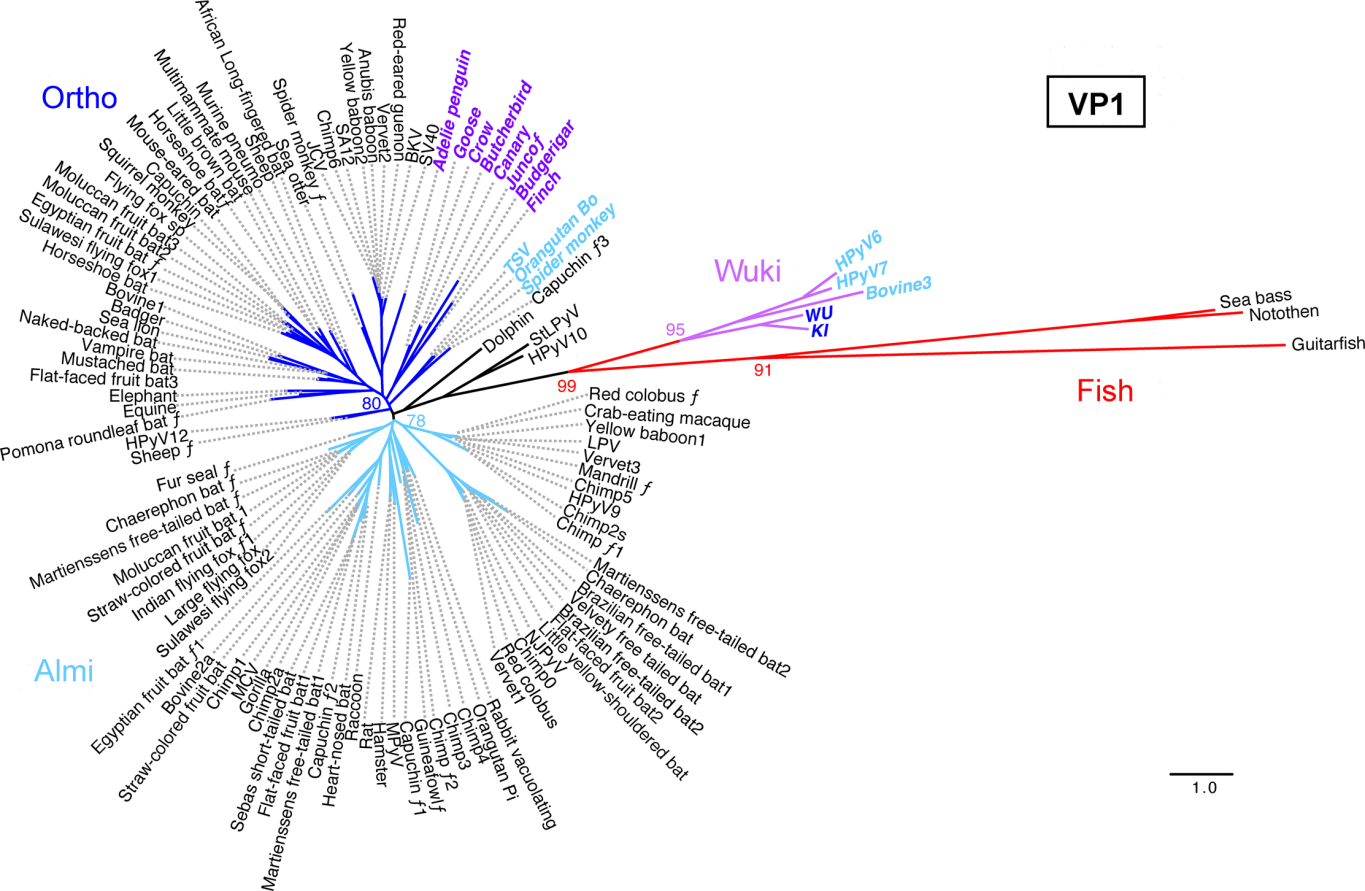
Midpoint-rooted phylogenetic tree for polyomavirus VP1 protein sequences. Species with different clade affiliations in LT analyses (Fig 3) are indicated in colored bold oblique text. The script ƒ character indicates fragmentary (sub-genomic) sequences. Percent bootstrap values for selected nodes are indicated. A FigTree file containing detailed bootstrap values is provided as S3 File. Scale bar shows one substitution per site.

Phylogenetic analyses of VP1 protein sequences (Fig 4) reveal somewhat different patterns. In contrast to avian polyomavirus LT protein sequences, avian polyomavirus VP1 sequences are interspersed among mammalian Ortho VP1 sequences. Phylogenetic analyses of VP2 protein sequences (presented in FigTree format in S4 File) are concordant with the VP1 analysis in this regard.

**Fig 5:**
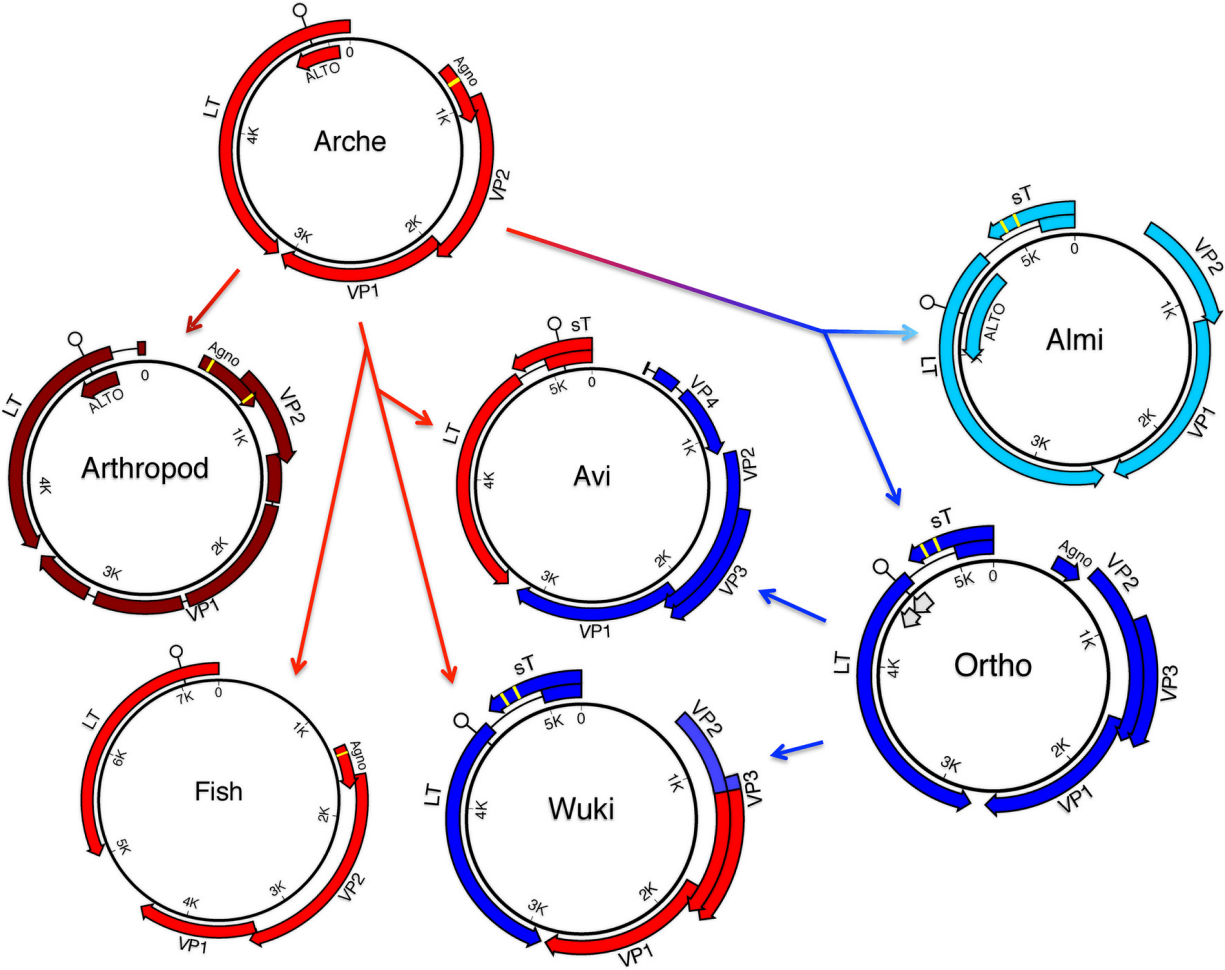
A hypothetical framework for ancient recombination events among major polyomavirus clades. The model attempts to reconcile observed incongruities between LT and VP1 phylogenetic trees shown in Figs 3 and 4. In the model, a hypothetical ancient polyomavirus, designated Arche, is inferred to have infected the last common ancestor of bilaterian animals. The ancient Arche lineage then gave rise to separate polyomavirus lineages found in arthropods and fish, as well as the mammalian Ortho/Almi lineages. The figure depicts Avi and Wuki clades arising after recombination events involving an unknown vertebrate-Arche lineage and Ortho-like species. The figure does not depict the inferred evolution of the HPyV6/7 clade, which appears to have arisen after a separate recombination event involving the late region of a hypothetical vertebrate-Arche lineage and the early region of a basal Almi-like species. The TSV lineage, which shows evidence of recombination between the Ortho and Almi lineages, is also omitted. White lollipops represent predicted pRb-binding motifs (LXCXE or related sequences). Yellow bars represent hypothetical metal-binding motifs (CXCXXC or related sequences). The absence of metal-binding motifs in Avi small T antigen (sT) proteins suggests a different evolutionary origin than the classic metal-binding Ortho/Almi sT. Possible ALTO-like ORFs predicted for some Ortho species are shaded gray.

Members of the previously recognized Wuki clade [29] encode VP1 protein sequences that occupy a highly divergent clade that distantly encompasses fish-associated VP1 sequences, while the early regions of Wuki species encode Ortho- or Almi-LT-like genes. Thus, relative to “classic” Ortho polyomaviruses the Avi clade shows a highly divergent early region while the Wuki clade shows a highly divergent late region. In Fig 5 we illustrate a recombination scheme that could account for this strangely mixed phylogeny.

### Accessory ORFs

The carboxy-terminal halves of Avi and bandicoot small T antigens (sT) show no linear sequence similarity to the sT proteins of Ortho or Almi polyomavirus species. In particular, Avi-type sT proteins lack highly conserved cysteine motifs that have recently been shown to coordinate iron-sulfur clusters in mammalian sT proteins [30]. It is also noteworthy that a conserved LXCXE motif (thought to be involved in interactions with the pRb family of tumor suppressor proteins and suppression of innate antiviral immunity [31]) is located on the shared sT/LT leader sequence in the Avi and bandicoot viruses, whereas the LXCXE motif is instead located in the second exon of LT in Ortho and Almi species. This suggests that Avi sT has a different evolutionary origin than Ortho/Almi sT. A possible explanation would be that Ortho/Almi sT arose after re-location of an ancestral cysteine motif-containing accessory gene into an N-terminal LT intron. For example, duplication of the scorpion polyomavirus ORF labeled “Agno” (Fig 1) into the LT intron could roughly reproduce an Ortho/Almi sT-like arrangement. A prediction of this idea would be that some of the hypothetical accessory ORFs of fish and arthropod polyomaviruses may be metal-binding proteins with Ortho/Almi sT-like functions, such as manipulation of cellular protein phosphatase 2A proteins [32].

ALTO is a recently discovered accessory gene that is “overprinted” in the +1 frame of the second exon of LT [27, 33, 34]. Although the function of ALTO is unknown, it shows sequence similarity to the C-terminal transmembrane domain of the well-studied middle T antigen of MPyV. This suggests that ALTO might, like middle T, function by mimicking activated growth factor receptors (reviewed in [35]). In their initial report demonstrating the existence of MCV ALTO, Carter et al. suggested that the gene might have first arisen in the Almi (ALTO/middle T) lineage after its divergence from the Ortho lineage. However, Carter and colleagues also noted that the ATG codon thought to initiate the translation of MCV ALTO and a hydrophobic sequence near the C-terminus of ALTO are partially conserved in other polyomaviruses outside the defined Almi clade. Puzzlingly, many recently discovered non-Almi polyomaviruses appear to have ALTO-like ORFs with lengths similar to some of the shorter examples of recognized Almi-LT ALTOs (summarized in S1 File). For example, the two variants of the scorpion polyomavirus potentially encode 9 or 13 kD ATG-initiated proteins in the +1 frame of the second exon of their LT sequences (see Fig 1). Despite the fact that the new supermarket sheep meat-associated polyomavirus occupies the Ortho clade, the +1 frame of its LT second exon encodes a potential 10 kD ALTO-like protein. One conceivable explanation for these observations might be that ALTO-like ORFs are an ancient ancestral feature that has been lost in some polyomavirus lineages. A possible example of occult or remnant ALTO/MT-like genes might be found in the small clade of primate polyomaviruses that encompasses SV40. Members of this group of viruses encode a short Met-initiated ORF in the +1 frame of the second exon of LT and a separate short downstream LT +1 frame ORF with a splice acceptor near its 5’ boundary (S3 Fig). It will be important to experimentally test the hypothesis that polyomavirus species outside the Almi-LT group express LT +1 frame ORFs as functional accessory proteins.

### Virus-host co-divergence

**Fig 6:**
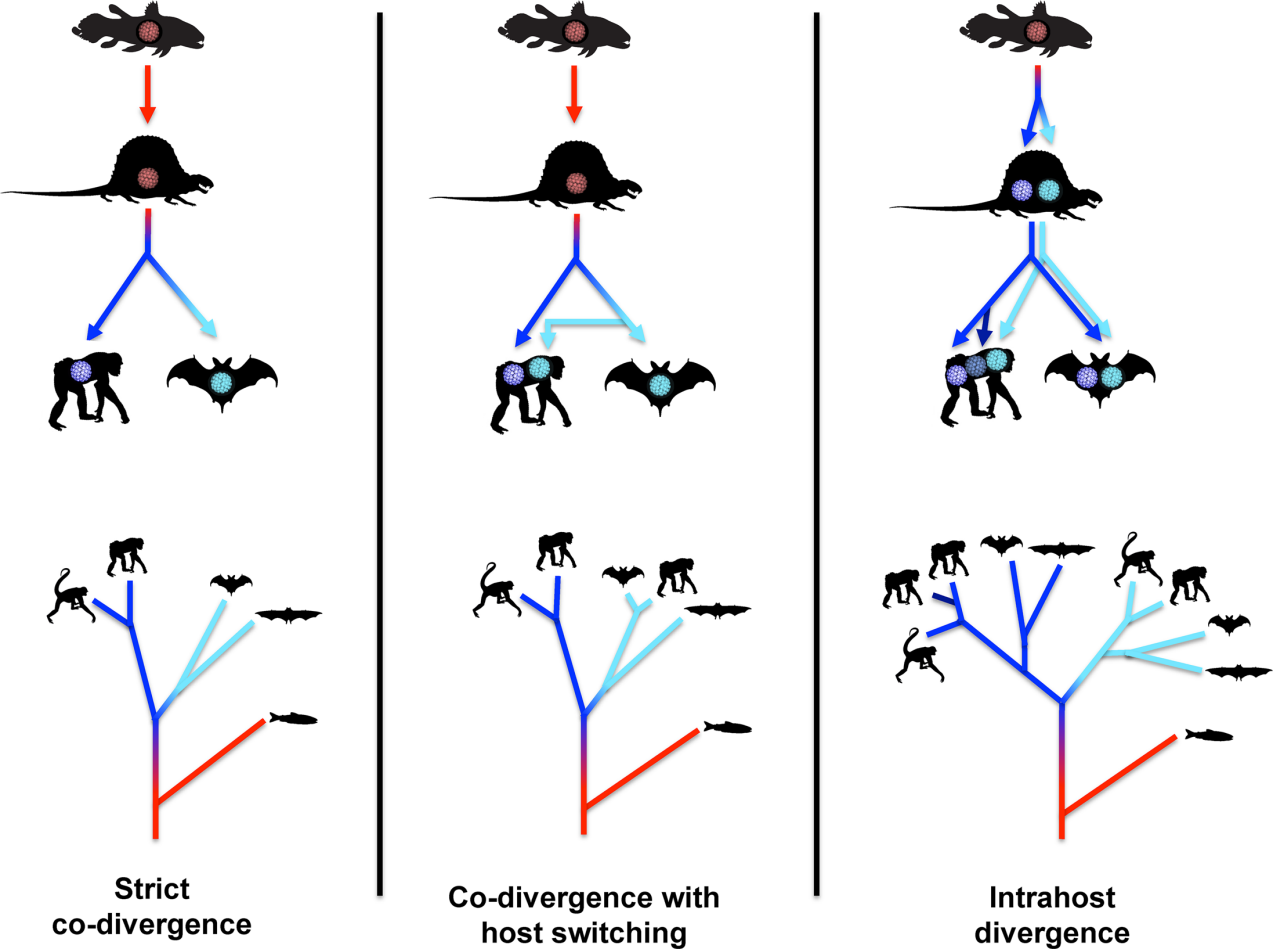
Standard virus/host co-divergence models. The top panels depict the evolution of polyomaviruses within animal lineages. Idealized cartoon trees in the bottom panels represent the expected polyomavirus phylogeny. The silhouettes in the bottom panels represent the animal type in which the polyomavirus at the branch tip would be found.

Three previously established [36–38] virus-host co-evolutionary models are summarized in simplified cartoon form in Fig 6. In the strict co-divergence model, the rate at which viruses “speciate” from one another exactly matches the rate at which host animals speciate. A group of retroviruses known as foamy viruses are an example of a viral genus that may at least roughly follow this evolutionary model [39]. Many prior studies have established that the family *Polyomaviridae*, as a whole, does not conform to the strict co-divergence model [37, 40–42].

In the co-divergence with host switching model (middle panel of Fig 6), viruses and hosts generally co-diverge, but viruses are occasionally productively transmitted between distantly related host animals. In the example, such events are reflected in finding closely related viral sequences in bats and great apes (see light blue branches). Ebola and influenza viruses are familiar examples of viruses with clear evidence of occasional long-range host switching.

**Fig 7:**
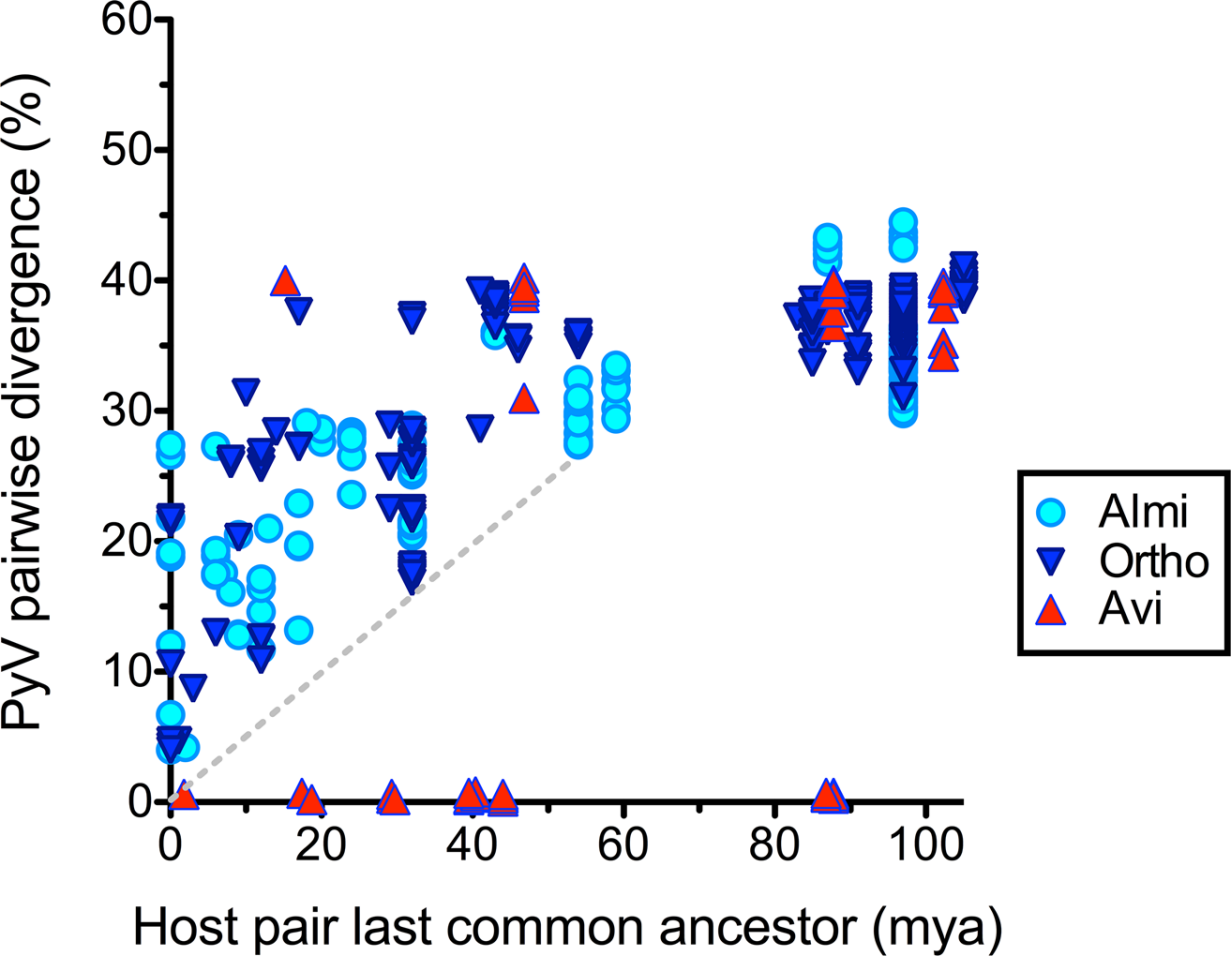
Virus-host co-divergence plot. SDT software was used to score individual pairs of polyomaviruses within various clades for percent divergence across the entire viral genome. The nucleotide divergence score was plotted against the estimated time (in millions of years ago, mya) of the last common ancestor of the host animals in which the polyomavirus pair was found. Apparent recent transmission of some Avi polyomaviruses between distantly related bird species is represented by points close to the x-axis. The absence of such points in the Almi and Ortho clades indicates a lack of evidence for recent transmission of polyomaviruses between distantly related mammal species. The arbitrary dashed reference line has a slope of about 0.5% polyomavirus divergence per million years after host divergence.

The first known polyomavirus of birds was discovered in diseased budgerigar fledglings (reviewed in [43]). Sequences >99% identical to the original budgerigar fledgling disease polyomavirus have subsequently been found in a surprisingly wide range of distantly related bird species [44–47]. Likewise, sequences nearly identical to goose hemorrhagic polyomavirus have been found in ducks [48, 49](accession JF304775). These prior findings are displayed as points close to the x-axis in Fig 7. Although the findings indicate that the host-switching model shown in the middle panel of Fig 6 might be applicable to some avian polyomaviruses, an important caveat is that all documented instances of inter-species Avi polyomavirus transmission have involved captive animals. It thus remains uncertain whether Avi polyomavirus host-switching occurs in the wild over longer timescales.

In contrast to Avi polyomaviruses, there are currently no examples of an individual polyomavirus species being found in more than one mammalian host (Fig 7). Most strikingly, there is no evidence of productive polyomavirus transmission between humans and any of the various polyomavirus-bearing animals we commonly live with or eat (i.e., budgerigars, canaries, geese, ducks, mice, rats, hamsters, capuchins, horses, cattle, sheep, caribou, or sea bass). The fact that the Rhesus macaque polyomavirus SV40 seems not to have gained a detectable foothold in the human population despite extremely widespread human exposure is also noteworthy in this regard. These observations suggest that the host-switching model is not generally applicable to mammalian polyomaviruses.

In the intrahost divergence model (right-hand panel of Fig 6), viruses diverge from one another at a faster rate than host animal speciation. Ancient viral divergence events occurring within a single host animal lineage eventually give rise to separate viral clades that co-occupy a single animal species. The model does not invoke transmission of viruses between distantly related host animals, but could accommodate viral transmission between closely related animal species or subspecies. In the shown example, the dark blue and light blue lobes of the viral phylogenetic tree each internally resemble the phylogeny of host animals. More recent intrahost viral divergence events are reflected as distinct but closely related viral species found within a single host animal (see dark blue branches). Herpesviruses and some retrovirus genera are well-documented examples of this form of viral evolution [50, 51].

The phylogeny of mammalian polyomavirus species is qualitatively similar to the intrahost divergence model. In particular, the two Almi “Monominor” sub-clades (defined as species that encode a recognizable large ALTO but lack VP3 [1, 27]) recapitulate the expected topology of the intrahost divergence model (S2–S5 Files, S4 Fig).

The intra-host divergence model predicts that homologs of polyomavirus species that occupy currently depauperate lobes of the tree, such as the small clade that encompasses only WU and KI, may ultimately be found in other mammals. This prediction is consistent with the recent discovery of two WU/KI-like polyomavirus species associated with two European vole genera [52] (see Discussion).

### Observed divergence analysis

A 2007 study by Carroll and colleagues showed that the VP1 nucleotide sequences of a panel MPyV strains found in feral mice collected in various locations in the United States all exactly matched the sequence of an MPyV laboratory isolate propagated in culture since 1953 [53]. Likewise, recent avian polyomavirus isolates are nearly identical to the isolate originally discovered in budgerigar fledglings in 1981 [54]. The concept that individual polyomavirus lineages may remain perfectly static over historical timescales is also consistent with the fact that BKV, JCV, MCV, and TSV strains with nearly or exactly identical nucleotide sequences have repeatedly been isolated from people residing on different continents [55, 56]. Historical sampling thus does not appear to be a tractable approach to measuring polyomavirus nucleotide sequence divergence rates. We set out to instead compare the observed divergence of different polyomavirus species to the estimated time of divergence of the host animals in which they were found. The analysis rests on the starting assumption that productive transmission of polyomaviruses between different mammal genera is rare or non-existent (Fig 7, S3 Fig, S5 File).

In the intrahost divergence model, distantly related polyomaviruses found in closely related animals reflect ancient polyomavirus divergence events that occurred long prior to the divergence of the host animal pair. Under this scenario, data points in the top left quadrant of Fig 7 would give an artificially fast estimate of the rate of polyomavirus sequence divergence. Despite this caveat, it seems reasonable to assume that polyomavirus divergence events might sometimes happen to coincide with host animal speciation events. This would be reflected as the lowermost Ortho and Almi points in the scatter plot shown in Fig 7. The arbitrary dashed line in the figure connects polyomavirus pairs that hypothetically happened to diverge from one another at about the same time that the host animal pair diverged. The slope of the line is consistent with the idea that at least some Ortho and Almi polyomavirus pairs cumulatively diverged by roughly 0.5% per million years, at least during the first 60 million years after divergence. This crude estimate is consistent with a more sophisticated phylogenetics-based Bayesian rate estimate by Krumbholz et al. of about 0.8% per million years (8 x 10^−9^ nucleotide substitutions per site per year) for the protein-coding segments of Ortho polyomaviruses [57]. A separate observed-divergence analysis of LT and VP1 proteins suggests that the two genes have independently accumulated non-silent changes at comparable long-term rates (S5 Fig). This rough result is also consistent with the more sophisticated prior work of Krumbholz and colleagues.

We also performed additional computational analyses to further confirm the prior rate estimates of Krumbholz et al. These analyses focused on the phylogenetically tractable Monominor clade. A ParaFit analysis of the clade as a whole indicates that the null hypothesis that polyomaviruses evolved independently of their hosts can be rejected, with a p-value of 0.0258. Based on the assumption that the separate Monominor A and B sub-clades arose after an ancient intrahost divergence event that pre-dated the first placental mammals, we performed separate ParaFit analyses on each Monominor sub-clade. These analyses indicate an even more confident rejection of the null hypothesis, with p-values of 1 x 10^−4^ and 8 x 10^−4^ for the A and B sub-clades, respectively. A BEAST analysis of concatenated LT and VP1 genes for the Monominor clade confirms that codon positions 1 and 2 evolve at a long-term rate of about 5 x 10^−9^ substitutions per site per year (i.e., 0.5% per million years), while codon position 3 evolves at a rate of about 2 x 10^−8^ substitutions per site per year. A time-resolved phylogenetic tree of the entire Monominor clade based on host phylogeny is shown in S5 File.

## Discussion

In this report, we propose a comprehensive theoretical framework for understanding the evolutionary history of the viral family *Polyomaviridae*. Our model suggests that the last common ancestor of arthropods and vertebrates harbored at least one polyomavirus. In the ensuing roughly half billion years, polyomaviruses appear to have accumulated genetic change at a remarkably slow cumulative long-term pace, in a pattern consistent with the intrahost divergence model diagrammed in Fig 6. Qualitative comparisons of phylogenetic trees suggest the occurrence of ancient recombination events involving distantly related polyomavirus species.

The intrahost divergence model also seems applicable to the evolution of papillomaviruses [58, 59]. A striking difference between polyomaviruses and papillomaviruses is the much greater number of known papillomavirus types. Our model could explain this difference simply by postulating that papillomaviruses evolve (and therefore undergo intrahost divergence) at a slightly faster rate than polyomaviruses. This is consistent with the findings of Rector and colleagues, who used phylogenetic analyses to estimate that papillomaviruses diverge at an observed long-term rate of 2% per million years (i.e., slightly faster than our phylogenetics-based long-term rate estimates for polyomaviruses)[57, 60].

In the current classification system approved by the International Committee on Taxonomy of Viruses (ICTV), all members of the family *Polyomaviridae* belong to a single genus, *Polyomavirus*. We have previously contributed to a proposal that the family be divided into three genera to be officially named *Orthopolyomavirus*, *Avipolyomavirus*, and *Wukipolyomavirus* [29]. A recent case study [61] helped us to appreciate a potential pitfall of the previously proposed taxonomic system. Clinical colleagues approached us about a lung transplant recipient whose lung-wash samples showed strong immunohistochemical reactivity with an antibody known to detect BKV and JCV LT proteins. Puzzlingly, the samples were negative for BKV and JCV by PCR. Although WU and KI were initially discovered in human respiratory samples [62, 63], we reasoned that the observed immunohistochemical staining was unlikely to represent cross-detection of WU or KI, since they occupy a different proposed genus than BKV and JCV. Hypothesizing that the sample might instead contain an undiscovered human polyomavirus related to BKV and JCV, we applied virion purification, random-primed RCA and deep sequencing methods. The deep sequencing revealed high levels of WU and no other polyomaviruses. With hindsight, we realize that a taxonomic system highlighting the close phylogenetic relationship between the LT proteins of BKV/JCV and WU/KI would have served us by suggesting the less time-consuming approach of performing simple WU/KI-specific PCR on the lung wash sample. In short, our failure to appreciate the now-apparent problem of inter-generic polyomavirus chimeras resulted in wasted effort.

An established taxonomic approach to the problem of chimerization is to separately categorize each major gene product. The most familiar example is the classification of influenza virus hemagglutinin (H) and neuraminidase (N) genes (e.g., H1N1, H5N1, etc.). As an example of applying this type of approach to polyomaviruses, BKV could be described simply as an Ortho species, while WU could be described as an Ortho-LT/Wuki-VP1 species. Like the influenza virus classification system, this form of nomenclature could serve as a colloquial set of conventions operating as an adjunct to official ICTV classifications (which can only be applied to entire organisms, as opposed to individual gene segments).

Our proposed colloquial classification scheme is in conflict with a recent formal proposal currently being considered by the ICTV. The new ICTV proposal suggests classifying polyomaviruses into four official genera based solely on the phylogeny of LT proteins [64]. Although the proposal is appealingly simple, it suffers from the “chimera-blindness” described in the case study above. For example, the proposal fails to recognize that all seven members of proposed genus *Gammapolyomavirus* encode VP1 and VP2 proteins that are monophyletic with proposed genus *Betapolyomavirus* VP1 and VP2 proteins. We suggest that the slightly greater complexity of the colloquial “flu-style” classification system proposed in the current study is justified by its greater taxonomic accuracy. Since the new four-genus proposal would awkwardly preclude the use of the more accurate flu-style classification, we concur with Tao and colleagues’ recent argument [41] in favor of preserving the existing ICTV standard, under which all polyomavirus species would officially remain in a single genus, *Polyomavirus*.

The intrahost divergence model predicts that multiple polyomaviruses with varying degrees of divergence will often be found within individual host animal species. Although we continue to favor the traditional cutoff of 81–84% identity across the entire viral genome for polyomavirus species distinctions, we note that this standard could be considered an arbitrary cutoff applied to a theoretically continuous variable. Since knowing the host animal species of origin appears to be of paramount importance for understanding polyomavirus evolution, we suggest that, in the future, it would be useful for new polyomavirus species names to reference the host animal species in which they were found. A possible problem with this approach is that, in some cases, a newly discovered virus might theoretically represent environmental contamination (as opposed to productive infection of the sampled animal). Our model provides a rough “back-of-the-envelope” approach to this question. As a concrete example, two recently discovered polyomavirus species whose genome sequences differ by about 20% were found separately in common voles and bank voles [52]. These two host species are thought to have diverged about 10 million years ago [65]. The two rodent-associated polyomaviruses differ from their nearest previously known relatives, human polyomaviruses WU and KI, by about 50%. Primates and rodents diverged about 90 million years ago [66]. Given the rough consistency of the observed divergences of the two new viruses with the >0.5% per million year “rule of thumb” shown in Fig 7, there seems to be no affirmative reason to suspect that the putative vole viruses originated in a non-rodent host. As polyomavirus phylogenetic trees become better populated, such guesswork could become increasingly confident.

It will be interesting to learn whether any un-recombined examples of the hypothetical vertebrate Arche lineage infect modern mammals. Since the chimeric bandicoot papilloma/polyomaviruses appear to carry an Arche-LT, it seems possible that Australian marsupials would be a promising group of animals in which to search for un-recombined Arche polyomaviruses. Similarly, a small Almi-VP1-like contig from the TSA dataset for helmeted guineafowl (*Numida meleagris*) raises the possibility that some modern birds may harbor un-recombined examples of the Almi clade. It will also be important to search for additional polyomavirus species in wild *Mus musculus*, as well as other common laboratory animals, such as zebrafish, sea urchin, *Caenorhabditis elegans*, *Xenopus laevis*, and *Drosophila melanogaster*. In addition to providing experimentally tractable models for exploring polyomavirus/host interactions, discovering new polyomavirus species in any of these animal lineages would shed additional light on the seemingly languid evolution of this fascinating family of viruses.

## Methods

### Sequence acquisition

Complete genome sequences of known polyomavirus species, as well as sub-genomic polyomavirus fragments, were downloaded from GenBank. Final database searches and downloads for sequences included in the shown phylogenetic analyses were performed on August 5, 2015. When necessary, the circular genome map was rearranged to comply with the convention that the initiator ATG of the Large T antigen (LT) CDS comprises the 5’ end of the antisense strand (see genome maps in Fig 1). In some instances, predicted splice sites or initiation codon annotations were altered based on alignments against other known polyomaviruses. MacVector 13 software was used to construct graphical maps.

Polyomavirus sequences that share <85% genome-wide pairwise nucleotide identity with other polyomaviruses are traditionally considered to be distinct viral species [29]. A few exceptions to the species cutoff rule were made for polyomavirus genomes with ≥85% identity that were isolated from different animal species. Examples of this exception include LPV/Vervet3 and Vervet2/Baboon2/SA12. Multiple representatives of each polyomavirus species were included in instances where different isolates with 85–95% identity could be found within the designated species. Each polyomavirus species was assigned a familiar nickname based on either a common name for the animal species with which it is associated or an established abbreviation (e.g., SV40, BKV, JCV).

As part of an ongoing *Trematomus* species physiology study in the Ross Sea, we sampled seven individual *Trematomus pennellii* (common name: sharp-spined notothen) caught using hook and line in McMurdo Sound during the summer field season of 2012–2013. *T*. *pennellii* are benthic nototheniid fish with a maximum body length of ~24 cm and are endemic to the Southern Ocean at a typical depth of ~ 1–100 meters. Their range can extend as far as ~700 meters [67]. Approximately 1 g of stomach, gills, liver and skin from the seven fish were grouped and each sample type was homogenized in 20 ml of SM buffer (0.1 M NaCl, 50 mM Tris/HCl–pH 7.4, 10 mM MgSO_4_) using a mortar and pestle, as previously described by Varsani et al. [68, 69]. Extracted DNA was sequenced on an Illumina HiSeq 2000 sequencer at Macrogen Inc. (South Korea) and the paired-end reads *de no*vo assembled using ABySS v1.5.2 [70] assembler (kmer = 64). In BLASTX [71] analyses, we identified a contig of ~6000 nt from the stomach sample that had similarity to polyomavirus LT. Based on this ~6000 nt *de novo* assembled sequence contig we designed abutting primers (PES-F: 5’-GTC GAC TTC TGT GCT GAC GTG ACT GAG-3’; PES-R: 5’-AGG TCC AGC CAT CTT CGG TGT ATC ACT T-3’) to recover the complete circular DNA molecule encompassing the LT-like sequence. Using the abutting primer pair with KAPA Hifi Hotstart DNA polymerase (Kapa Biosystems, USA) we amplified the polyomavirus-like circular molecule using the following protocol: initial denaturation at 95°C for 3 min followed by 25 cycles at 98°C for 20 sec, 60°C for 15 sec, 72°C for 5min and a final extension at 72°C for 5min. We were able to recover the ~6 kb amplicon from the liver and the stomach samples and these were cloned into pJET1.2 plasmid (ThermoFisher, USA), and Sanger-sequenced by primer walking at Macrogen Inc. (Korea). The Sanger-sequences were assembled using DNAbaser v.4 (Heracle BioSoft S.R.L., Romania). The complete genome of sharp-spined notothen (*Trematomus pennellii*) polyomavirus 1 (6219 nt) was 100% identical in both the stomach and liver deep sequencing samples and has been deposited in GenBank (accession KP768176).

Giant guitarfish (*Rhynchobatus djiddensis*) polyomavirus 1 (GenBank accession KP264963) was detected using previously reported methods [72] in specimens from an aquarium animal suffering from proliferative skin lesions. The guitarfish polyomavirus was discovered alongside much higher levels of a member of a different DNA virus family. The sequence of the other virus, and details on the pathology of the guitarfish specimen, will be published in a separate report.

Previously reported methods were used to discover sheep (*Ovis aries*) meat-associated polyomavirus 1 (GenBank accession KP890267) in a sample of ground lamb meat purchased at a US supermarket [18].

Baja California bark scorpion (*Centruroides exilicauda*) polyomavirus 1 was initially identified in a TBLASTN [71] search of the NCBI Whole Genome Shotgun database (WGS) using the LT protein sequence of black sea bass polyomavirus as bait. A single contig, accession number AXZI01204118, was curated back to the original reads (Sequence Read Archive (SRA) accession number SRX476227). The back-curation revealed that small segments were missing from the ends of the original contig. The SRA dataset contained at least three distinct viral sequence variants. The two most abundant variants were compiled separately. The putative LT intron (where the original contig ends fell) was an apparent polymorphic hotspot. The extensive variation in this portion of the polyomavirus genome could explain why the contig assembly process failed at this particular point. No chimeric reads (potentially representing integration of the viral genome into the host animal’s DNA) were detected, suggesting that both viral genomes were carried in an episomal form. Because current GenBank policies do not allow deposits of third-party sequence assemblies, the two scorpion polyomavirus sequences were instead deposited at EMBL (accession numbers LN846618 and LN846619).

### Abbreviation and naming conventions

In the interest of clarity, this manuscript favors the use of host animal common names and avoids the extensive use of abbreviations. In our view, when abbreviations are necessary they should be short, easily inferred as representing the host animal species of origin, and, ideally, should serve as pronounceable “sigla” http://ictvonline.org/codeofvirusclassification_2012.asp. We suggest that newly coined abbreviations should use a condensation of a common name for the host animal and “PyV” for polyomavirus. Examples of pronounceable abbreviations might be ShePyV1 for supermarket sheep meat-associated polyomavirus 1 or ChimPyV1 for *Pan troglodytes verus* polyomavirus 1.

Possible accessory proteins were detected by analyzing genome sequences for ORFs of at least 25 codons. Small T antigen (sT) was defined as an ORF encoding an ATG-initiated protein of at least 10 kD near the 5’ end of the LT gene. ALTO was defined as a >250 bp ATG-initiated ORF in the LT +1 frame located near the 5’ end of the LT exon encoding the helicase domain. In nearly all cases, the ALTO ORF overlaps the segment of LT encoding the putative pRb-interaction motif LXCXE. Agno was defined as an ORF encoding a >10 kD protein initiated from an ATG codon located upstream of the inferred VP2 ORF.

An attempt was made to infer the LT-binding sites associated with the viral origin of replication. The “classic” Oris of SV40 and MPyV were defined as paired palindromic GRGGCY motifs adjacent to an A/T tract. Hypothetical Avi and fish Ori sequences were defined as paired palindromic YYTGSCA motifs adjacent to an A/T tract. A hypothetical arthropod Ori was defined as paired palindromic ATCACGYG motifs flanked on both sides by A/T tracts.

### Structural modeling

The analyses of the Large T antigens (LTs) from scorpion, guitarfish and notothen polyomaviruses were performed using multiple bioinformatics tools from the psipred server, http://bioinf.cs.ucl.ac.uk/psipred/?disopred=1 [73]. In order to obtain models of high quality, the structural relationships between the novel LTs and previously solved protein structures were determined through fold recognition using pGenTHREADER and pDomTHREADER from the psipred server [74]. Matching structures with the highest scores were then selected as templates for predicting structures of the novel LTs. Models for DNAJ and OBD-Zn-ATPase were generated separately. All structures and models were visualized and compared using PyMOL (The PyMOL Molecular Graphics System, Version 1.2r3pre, Schrödinger, LLC).

MEME suite 4.10.0 http://meme.nbcr.net/meme/ [75] was used to facilitate the identification of possible palindromically arranged LT-binding motifs in candidate Ori regions. Inferred candidate motifs are indicated in the legend of Fig 5.

### Phylogenetic analyses

Curated polyomavirus sequence sets used in this work are posted at http://home.ccr.cancer.gov/Lco/PyVE.asp. The site includes annotated genomes for examples of all currently known polyomavirus species and compiled protein sequences.

Initial exploratory phylogenetic analyses were performed using the Phylogeny.fr website http://phylogeny.lirmm.fr/ in “One Click” mode without Gblocks [76]. FigTree software v1.4.2 http://tree.bio.ed.ac.uk/software/figtree/ was used to display trees. Confirmatory analyses were performed by aligning sequences using MUSCLE [77] and manually editing the output. Maximum-likelihood phylogenetic trees (with approximate likelihood branch support, aLRT) were inferred using PHYML 3 [78] with LG+I+G as the best substitution model determined using ProtTest [79]. Branches with <80% aLRT branch support were collapsed. Confirmatory Bayesian phylogenetic analyses showed essentially identical tree topology. However, Bayesian phylogenetic trees for VP1 proteins showed poor support values. The results are consistent with a pending ICTV proposal http://talk.ictvonline.org/files/proposals/animal_dna_viruses_and_retroviruses/m/animal_dna_under_consideration/5637.aspx. Because of their better bootstrap values, maximum-likelihood analyses were favored for the current study.

Nucleotide divergence calculations were performed for individual sequence pairs using Sequence Demarcation Tool (SDT) version 1.2 in MUSCLE mode [80, 81] http://web.cbio.uct.ac.za/~brejnev/. Pairwise calculations were performed on discrete clades, specifically: the separate “Monominor” A and B sub-clades, the Ortho-LT clade (excluding WU and KI), the “Blympho” clade (which houses B-lymphotropic polyomavirus (LPV) and HPyV9), and the two small clades that separately house TSV and Chimp3. For Avi polyomaviruses, sequences found in the “fragments” tab of S1 File were included in the analysis. The analysis was performed in January 2015 and does not include polyomavirus sequences made public after that time.

Estimates of the time to last common ancestor of animal species pairs were based on various references [82–87]. In most cases, the estimates were based primarily on sequence analyses, as opposed to fossil records. Estimates are consistent (to within 10%) with the “Expert Result” in Time Tree of Life http://www.timetree.org/ [65].

### Test of polyomavirus and host co-speciation

To ensure maintenance of codon information, nucleotide sequences for the VP1 and Large T coding regions were translated into protein sequences. The translated proteins were aligned using Mafft (implementing the L-ins-I algorithm)[88]. Next, the aligned protein sequences were reverse translated into nucleotide sequences. Finally, the individual alignments were concatenated into a supermatrix.

To test for potential substitutional saturation [89, 90] the index of substitutional saturation statistic was calculated for the supermatrix (test implemented in DAMBE version 6.0.0 [91]). The results indicated that the observed saturation index of 0.5865 was smaller than the critical saturation index (Iss.c = 0.8023), suggesting that the sequences have experienced little substitutional saturation, thus conserving sufficient phylogenetic signal for phylogenetic reconstruction.

PartitionFinder v1.1.1 was used to select the best-fit partitioning schemes and partition-specific substitution models under the Bayesian information criterion (BIC) [92]. PartitionFinder suggested the use of 4 different partitions [(Large T codon position 1, VP1_CP1), (Large T_CP2, VP1_CP2), (VP1_CP3), and (Large T_CP3)]. All partitions were estimated to evolve under the General Time Reversible (GTR) model of nucleotide substitution with invariant sites (I) and Γ distributed rate variation among sites (GTR+I+G).

Parafit was used to formally test the hypothesis of coevolution between Monominor polyomaviruses and their associated hosts [93, 94]. The null hypothesis (H0) of the global test is that the evolution of polyomavirus species and the host animals in which they were found has been independent. The test, as implemented within the R package (APE) version 3.3 [95] requires two phylogenetic trees and the set of host-parasite association links. The host tree was constructed using phyloT (available from http://phylot.biobyte.de/). PhyloT uses NCBI taxonomy identification numbers to generate a phylogenetic tree. The obtained tree was manually edited to include branch lengths of unit length. MrBayes 3.2.6 [96, 97], as implemented within the CIPRES Science Gateway V. 3.3 [98], was used to estimate the Monominor phylogenetic tree. The selected GTR+I+G substitution model was implemented. The analysis was run using two independent chains for a total chain length of one million iterations, with a sampling frequency every 1,000^th^ step. Following a 10% burn-in, the tree was summarized. The GlobalParafit was estimated to be 3633.384, with a p-value = 0.0258 (based on 1,000 permutations), providing support in favor of co-speciation.

### Estimation of the evolutionary rate of the Monominor clade

The supermatrix described in the previous section was used for this analysis.

The Bayesian analysis (Beast 1.8 [99]) as implemented within the CIPRES Science Gateway V. 3.3 [98], was performed using linked substitution rates for the first and second codon positions (CP_12_), while allowing independent rates in CP_3_. The uncorrelated lognormal relaxed molecular clock was used to accommodate rate variation among lineages. Monophyletic constraints were placed on the separate Monominor A and B clades. Based on the posterior distributions obtained for the host [84, 100], normal priors were imposed on specific nodes used to calibrate the evolutionary rates (S5 File). Three independent Markov Chain Monte Carlo (MCMC) analyses were run for 10 million generations each, with samples from the posterior drawn every 1,000 generations. The first 10% of each run was discarded prior to the construction of the posterior probability distributions of parameters. Each analysis was run sufficiently long that effective sample sizes for parameters were >400. The results from the three runs were combined to generate a maximum clade credibility tree and rate and divergence time summaries (S5 File).

## Supporting Information

S1 Fig In situ hybridization analysis of guitarfish polyomavirus in resolving skin lesions. A hybridization assay adapted from previously reported methods [101, 102] was used to stain sections of guitarfish skin lesions biopsied during the resolution of symptoms. Guitarfish polyomavirus VP1 probe hybridization signal (red) was observed in unidentified round cells. Arrows indicate selected positively-stained cells. The cells appear to have histiocytic or macrophage-like morphology. Free speckled brown/black patterns are attributable to melanin. Scale bar represents 20 μm.
(TIF)

S2 Fig Conservation maps for LT DNAJ and Zn-ATPase domains. The conservation maps were generated using the ConSurf server (http://consurf.tau.ac.il/), and then visualized using Chimera, http://www.cgl.ucsf.edu/chimera/ [103]. Panel A: the DNAJ domain conservation map was generated using DNAJ domain sequences from 34 polyomavirus LTs in the Uniref90 collection. The black oval indicates the highly conserved HPDKGG motif. Panel B: conservation map of LT Zn-ATPase domains. The map was generated with 69 LT sequences from the Uniref90 collection. The black oval indicates the Walker motifs required for binding and hydrolysis of ATP. Fewer DNAJ domains were included in this analysis due to a stringent default E-value (0.0001) setting. This indicates a greater level of variation among the DNAJ domains in contrast to the Zn-ATPase domains of LTs.
(TIF)

S3 Fig Analysis of LT +1 frame ORFs. The genome map depicts BKV-I as a representative example of the small clade of primate polyomaviruses encompassing SV40.
(TIF)

S4 Fig Phylogenetic illustration of select pairwise divergences. Phylogeny.fr “one click” settings were used to draw a phylogenetic tree for the complete genomes (nucleotide) of selected members of the Almi-LT and Ortho-LT clades. The tree is arbitrarily rooted on human polyomavirus 9. The selected Almi species have only one minor capsid protein and thus belong to a “Monominor” sub-clade within clade Almi. Numbers within the nodes indicate the estimated time (in millions of years ago) of the last common ancestor of host animals contained within the node. Branches are color-coded based on host animal families. Percentages indicate the pairwise nucleotide divergence of the complete genomes of the indicated polyomavirus species pair. Nodes that encompass possible intra-host polyomavirus divergence events are marked with asterisks.
(TIF)

S5 Fig LT and VP1 co-divergence. SDT was used to calculate the percent divergence of LT and VP1 proteins for individual pairs of polyomaviruses. The linear relationship between LT and VP1 divergences in Ortho, Almi, and fish clades suggests that the two proteins independently diverge at a roughly similar rate. The disconnection of the Avi and Wuki clades can most easily be explained by ancient recombination events (see Fig 5).
(TIF)

S1 File Naming key. (XLSX)

S2 File LT phylogenetic tree (FigTree format http://tree.bio.ed.ac.uk/software/figtree/). (TRE)

S3 File VP1 phylogenetic tree (FigTree format). (TRE)

S4 File VP2 phylogenetic tree (FigTree format). (TRE)

S5 File Time-resolved phylogenetic tree of the Monominor polyomavirus clade. Tabular data refers to the numbered nodes in the phylogenetic tree. The table indicates the posterior probability, node age (including 95% HPD), average (95% HPD) rate for each partition, and presence of constraints for individual nodes. The phylogenetic tree displays the evolutionary relationship between members of the Monominor clade. The tree and geological column were generated using the (APE) package within R. The scale bar indicates millions of years before the present. The inset shows the median evolutionary rate (with 95% HPD) of the 1^st^-2^nd^ and 3^rd^ codon positions.
(XLSX)
