# Supplementary figures and images for: The Ancient Evolutionary History of Polyomaviruses
Source: PLoS Pathog. 2016 Apr 19;12(4):e1005574. doi: 10.1371/journal.ppat.1005574 (PMC4836724; doi:10.1371/journal.ppat.1005574)

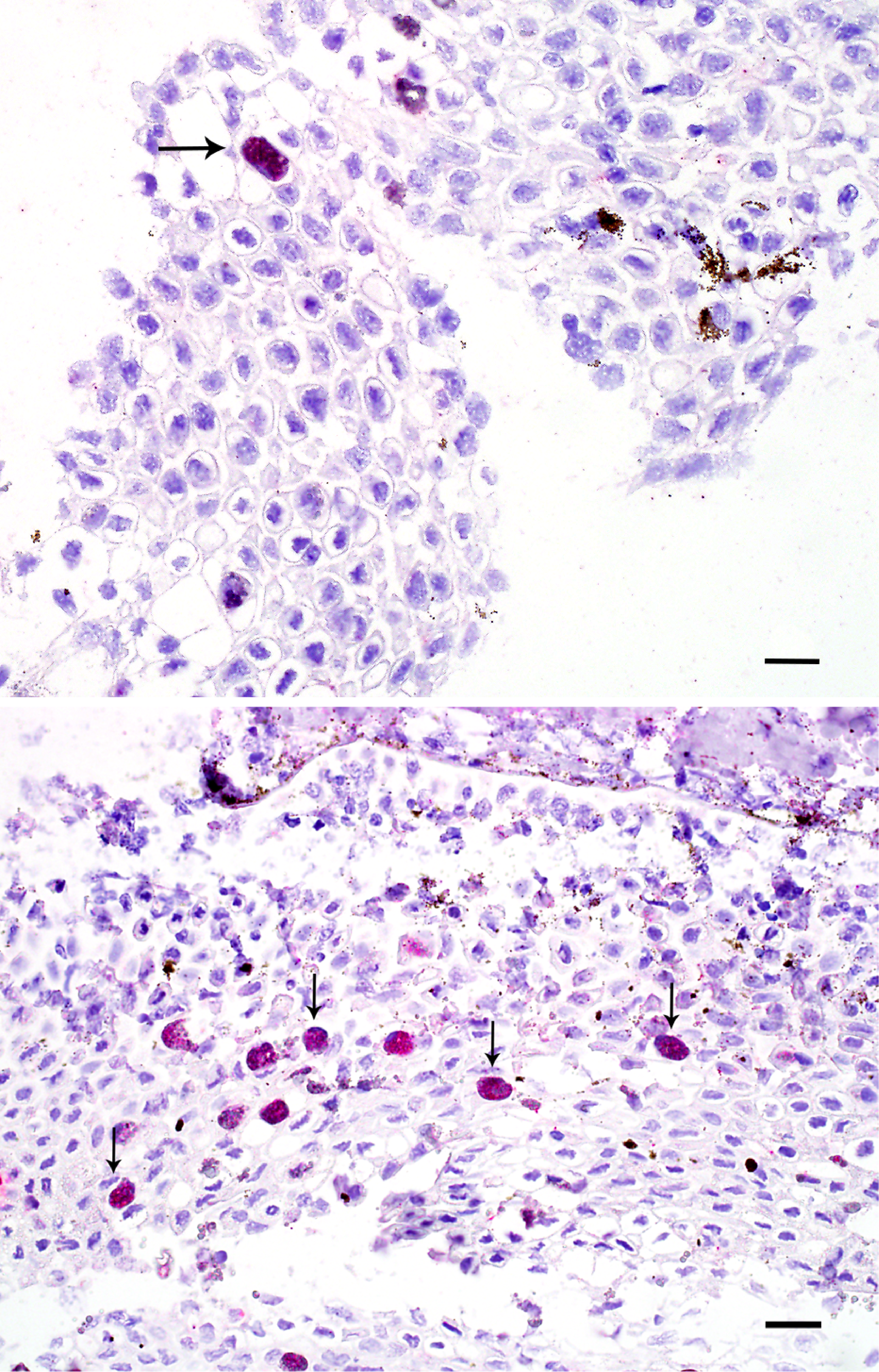

Supplement: S1 Fig — A hybridization assay adapted from previously reported methods [101, 102] was used to stain sections of guitarfish skin lesions biopsied during the resolution of symptoms. Guitarfish polyomavirus VP1 probe hybridization signal (red) was observed in unidentified round cells. Arrows indicate selected positively-stained cells. The cells appear to have histiocytic or macrophage-like morphology. Free speckled brown/black patterns are attributable to melanin. Scale bar represents 20 μm. (TIF) [file ppat.1005574.s001.tif]

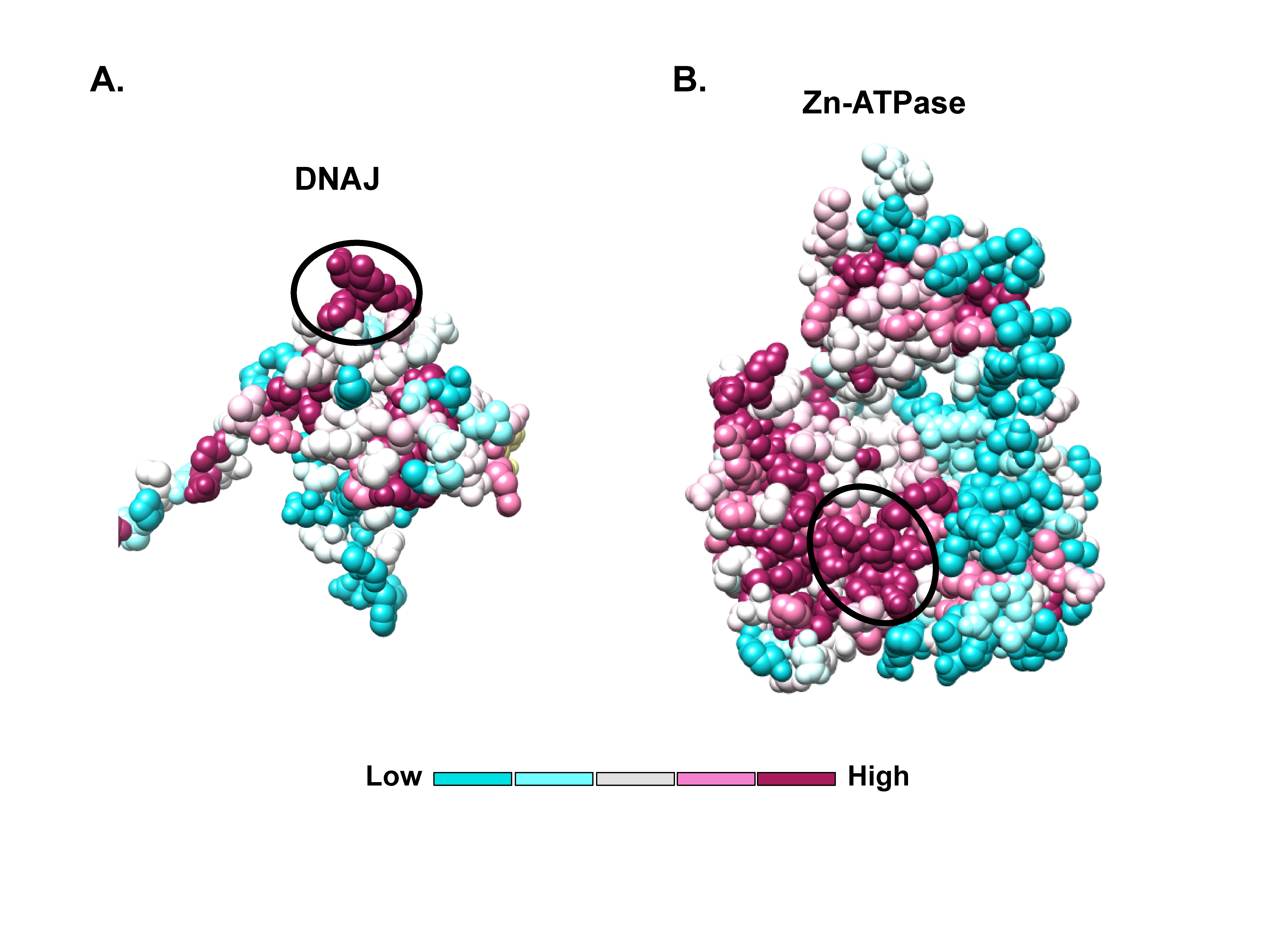

Supplement: S2 Fig — The conservation maps were generated using the ConSurf server (http://consurf.tau.ac.il/), and then visualized using Chimera, http://www.cgl.ucsf.edu/chimera/ [103]. Panel A: the DNAJ domain conservation map was generated using DNAJ domain sequences from 34 polyomavirus LTs in the Uniref90 collection. The black oval indicates the highly conserved HPDKGG motif. Panel B: conservation map of LT Zn-ATPase domains. The map was generated with 69 LT sequences from the Uniref90 collection. The black oval indicates the Walker motifs required for binding and hydrolysis of ATP. Fewer DNAJ domains were included in this analysis due to a stringent default E-value (0.0001) setting. This indicates a greater level of variation among the DNAJ domains in contrast to the Zn-ATPase domains of LTs. (TIF) [file ppat.1005574.s002.tif]

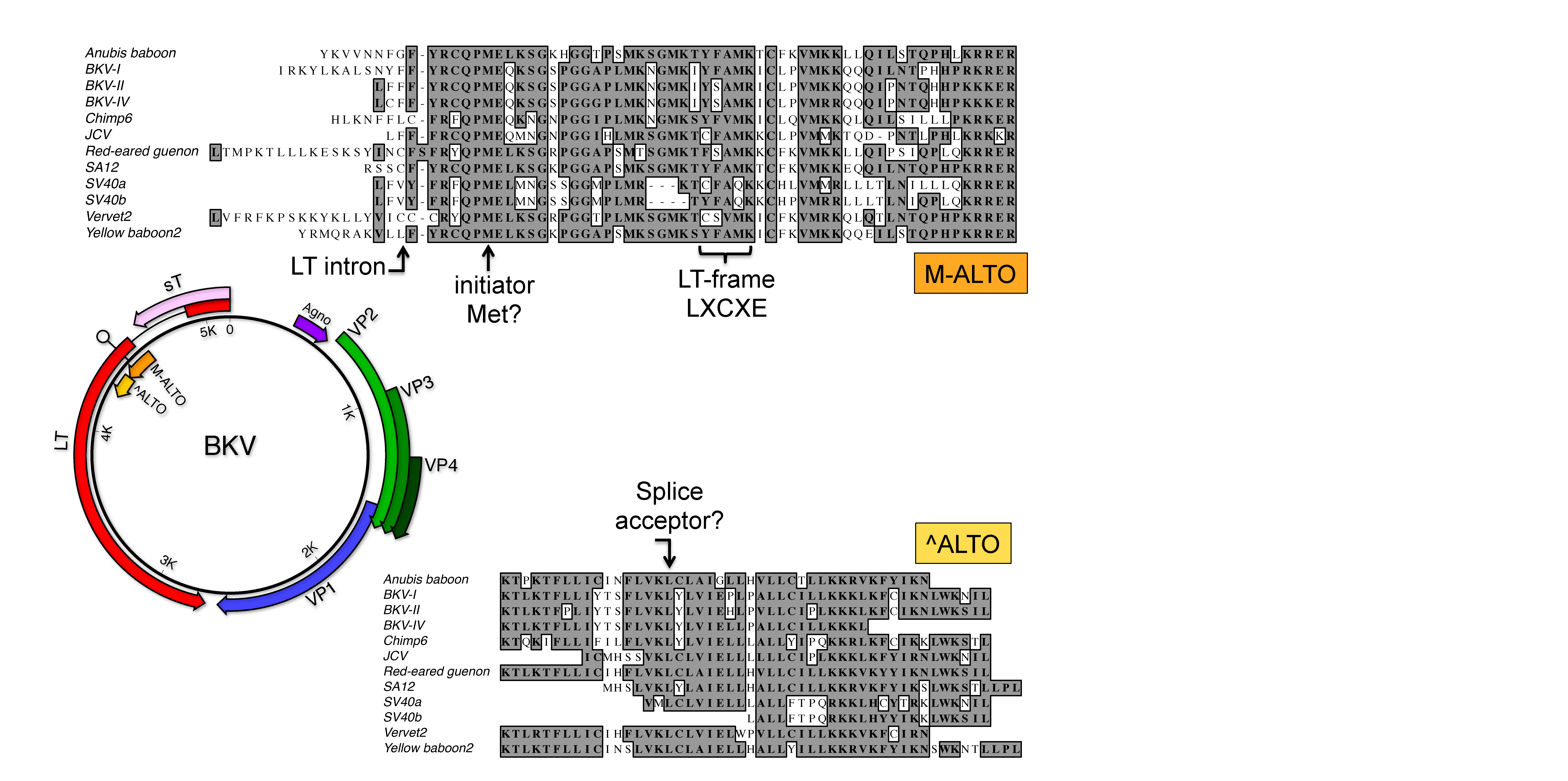

Supplement: S3 Fig — The genome map depicts BKV-I as a representative example of the small clade of primate polyomaviruses encompassing SV40. (TIF) [file ppat.1005574.s003.tif]

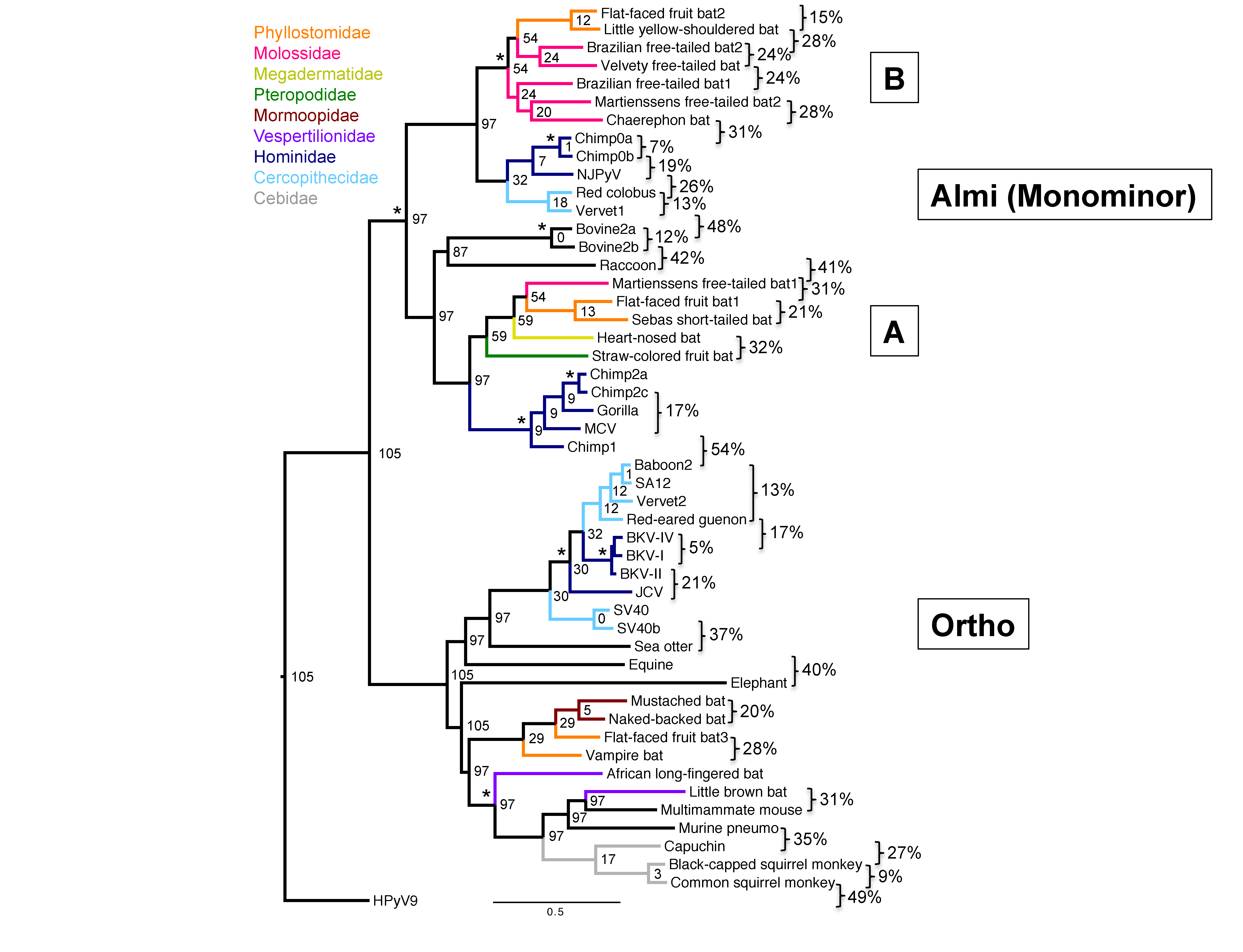

Supplement: S4 Fig — Phylogeny.fr “one click” settings were used to draw a phylogenetic tree for the complete genomes (nucleotide) of selected members of the Almi-LT and Ortho-LT clades. The tree is arbitrarily rooted on human polyomavirus 9. The selected Almi species have only one minor capsid protein and thus belong to a “Monominor” sub-clade within clade Almi. Numbers within the nodes indicate the estimated time (in millions of years ago) of the last common ancestor of host animals contained within the node. Branches are color-coded based on host animal families. Percentages indicate the pairwise nucleotide divergence of the complete genomes of the indicated polyomavirus species pair. Nodes that encompass possible intra-host polyomavirus divergence events are marked with asterisks. (TIF) [file ppat.1005574.s004.tif]

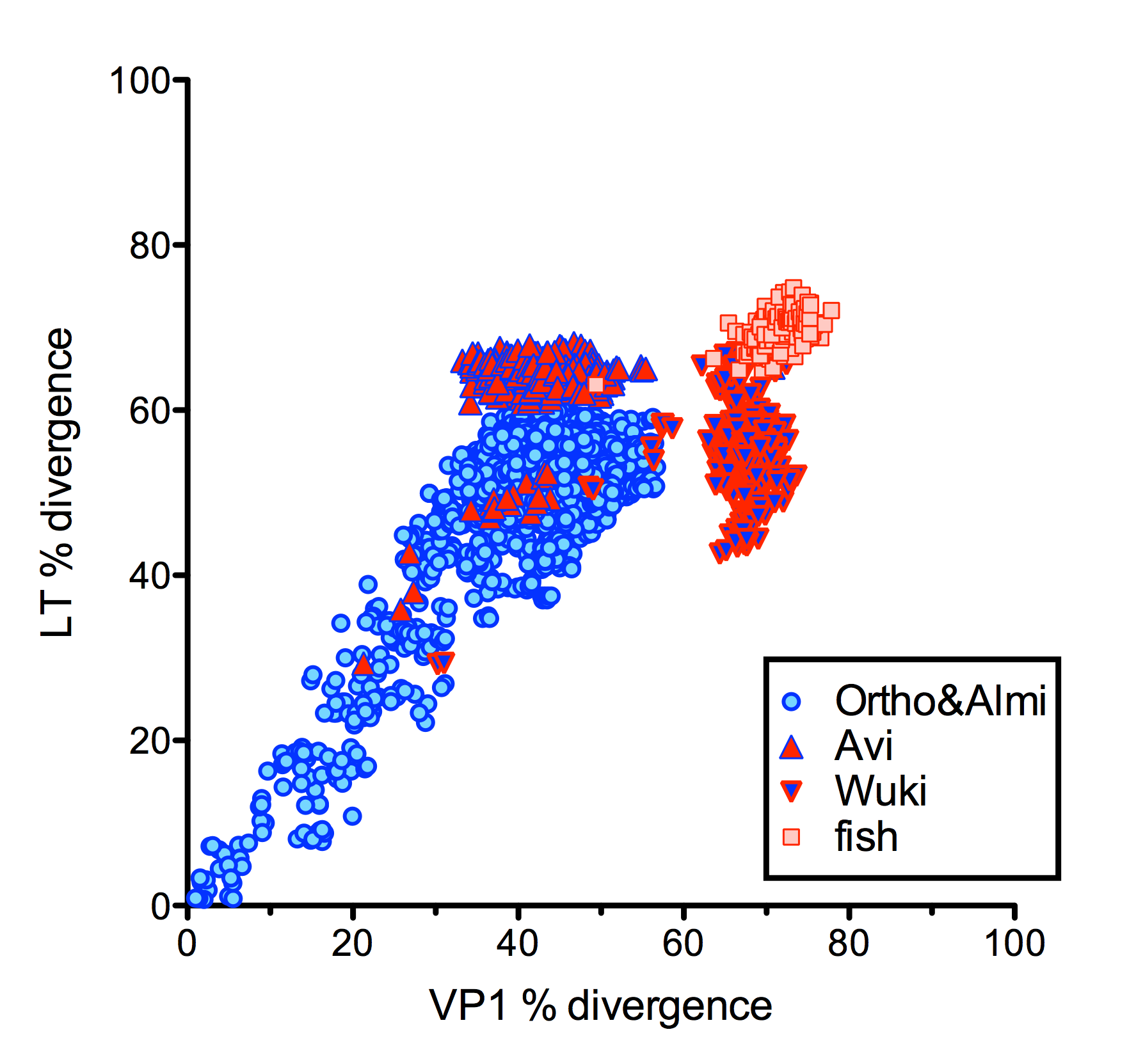

Supplement: S5 Fig — SDT was used to calculate the percent divergence of LT and VP1 proteins for individual pairs of polyomaviruses. The linear relationship between LT and VP1 divergences in Ortho, Almi, and fish clades suggests that the two proteins independently diverge at a roughly similar rate. The disconnection of the Avi and Wuki clades can most easily be explained by ancient recombination events (see Fig 5). (TIF) [file ppat.1005574.s005.tif]
